# Decline in Clinical Efficacy of Oral Miltefosine in Treatment of Post Kala-azar Dermal Leishmaniasis (PKDL) in India

**DOI:** 10.1371/journal.pntd.0004093

**Published:** 2015-10-22

**Authors:** V. Ramesh, Ruchi Singh, Kumar Avishek, Aditya Verma, Deepak Kumar Deep, Sandeep Verma, Poonam Salotra

**Affiliations:** 1 Dermatology Department, Safdarjung Hospital and Vardhman Mahavir Medical College (VMMC), New Delhi, India; 2 National Institute of Pathology (ICMR), Safdarjung Hospital Campus, New Delhi, India; Institute of Post Graduate Medical Education and Research, INDIA

## Abstract

**Background:**

Recent studies have shown significant decline in the final cure rate after miltefosine treatment in visceral leishmaniasis. This study evaluates the efficacy of miltefosine in the treatment of post kala-azar dermal leishmaniasis (PKDL) patients recruited over a period of 5 years with 18 months of follow-up.

**Methodology:**

In this study 86 confirmed cases of PKDL were treated with two different dosage regimens of miltefosine (Regimen I- 50mg twice daily for 90 days and Regimen II- 50 mg thrice for 60 days) and the clinical outcome assessed monthly. Cure/relapse was ascertained by clinical and histopathological examination, and measuring parasite burden by quantitative real-time PCR. *In vitro* susceptibility of parasites towards miltefosine was estimated at both promastigote and amastigote stages.

**Results:**

Seventy three of eighty six patients completed the treatment and achieved clinical cure. Approximately 4% (3/73) patients relapsed by the end of 12 months follow-up, while a total of 15% (11/73) relapsed by the end of 18 months. Relapse rate was significantly higher in regimen II (31%) compared to regimen I (10.5%)(P<0.005). Parasite load at the pre-treatment stage was significantly higher (P<0.005) in cases that relapsed compared to the cases that remained cured. *In vitro* susceptibility towards miltefosine of parasites isolated after relapse was significantly lower (>2 fold) in comparison with the pre-treatment isolates (P<0.005).

**Conclusion:**

Relapse rate in PKDL following miltefosine treatment has increased substantially, indicating the need of introducing alternate drugs/ combination therapy with miltefosine.

## Author Summary

Increasing resistance to antimonials has paved the way for the oral drug miltefosine for PKDL treatment. Recent studies show a significant decline in the final cure rate of VL after miltefosine treatment in the Indian subcontinent. This is the first study to evaluate the efficacy of miltefosine treatment in a large number of PKDL cases with 18 months follow-up. PKDL cases that completed miltefosine treatment responded well and showed initial cure; however, with 18 months of follow up period, the final cure rate was only about 85%. Treatment regimen based on high dosage over short duration resulted in high relapse rate. We observed that the parasites isolated from the cases that relapsed were more tolerant to miltefosine (>2 fold) compared to the isolates from pre-treatment stage. Estimation of parasite load at pre-treatment stage indicated that the patients with higher initial parasitic burden were at a higher risk of relapse. The declining efficacy of monotherapy with miltefosine warrants the need of alternate regimens for treatment of PKDL.

## Introduction

Post kala-azar dermal leishmaniasis (PKDL), a dermatosis that usually develops as a sequealae of visceral leishmaniasis (VL) or kala-azar (KA), is reported mainly in East Africa and the Indian sub-continent [1]. The disease appears as a macular, papular or nodular rash, or a combination of these, frequently on the face followed by the trunk and limbs. It may also affect the conjunctival, nasal, oral and genital mucosa. PKDL occurs approximately in 5–15% and 50–60% of apparently cured cases of VL in the Indian subcontinent and Sudan respectively and is considered an important parasite reservoir for the transmission of disease along with VL patients [1]. Thus, adequate monitoring and treatment of PKDL constitutes key importance in the ongoing effort to eliminate VL.

Standard treatment of PKDL remains unsatisfactory. Miltefosine (MIL) is the only orally effective antileishmanial drug, which was first approved for VL treatment in India in 2002 [2]. It has been found to be well tolerated and effective for PKDL with a cure rate of 78% by ITT analysis and 93% by protocol analysis in monotherapy and in combination with amphotericin B (AmB) [3, 4] However, due to its long half life, this drug is highly susceptible to the development of resistance. Recent studies on treatment of the Indian VL with MIL showed a significant decline in the final cure rate from 94% (phase III trial during 1999–2000) to approx 90%, with a doubling of relapse rate from 3% to 6.8% after a decade of its use [4]. Similarly, the increase in relapse rate from 10 to 20% in long term follow up of 12 months compared to 6 months post MIL treatment was observed in Nepalese patients [5].

Previously, the *in vitro* susceptibility of *L*. *donovani* isolates from PKDL lesions was shown to be significantly lower in isolates obtained from relapse cases after MIL treatment in comparison with the pre-treatment isolates [6]. It was suggested that the reduced drug susceptibility of these isolates may be due to longer treatment regime leading to prolonged exposure of parasites to the drug, as observed in the case of sodium antimony gluconate (SAG) unresponsiveness in PKDL [7]. The emerging picture with MIL is similar to what has already been seen in the case of antimonials, where initially there was a gradual increase in tolerance to the drug, followed by reduced efficacy and finally the development of resistance. Therefore, effective monitoring of the valuable antileishmanial drug MIL is imperative in order to prevent the emergence of drug resistance.

The present article describes the clinical efficacy of MIL treatment in the Indian PKDL in a large number of patients hailing from areas representative of the entire endemic region in India, with a long term follow up of 18 months. *In vitro* susceptibility of PKDL isolates towards MIL and the parasite burden in dermal lesions of cases that relapsed have been compared with the cases that remained cured.

## Materials and Methods

### Ethics statement

The study was approved by the Ethical Committee of SJH and written informed consent was obtained from the patients or their guardians. Additionally, relapse case no. 11 gave written consent for publication of his photograph.

### Study patients and treatment

**Fig 1:**
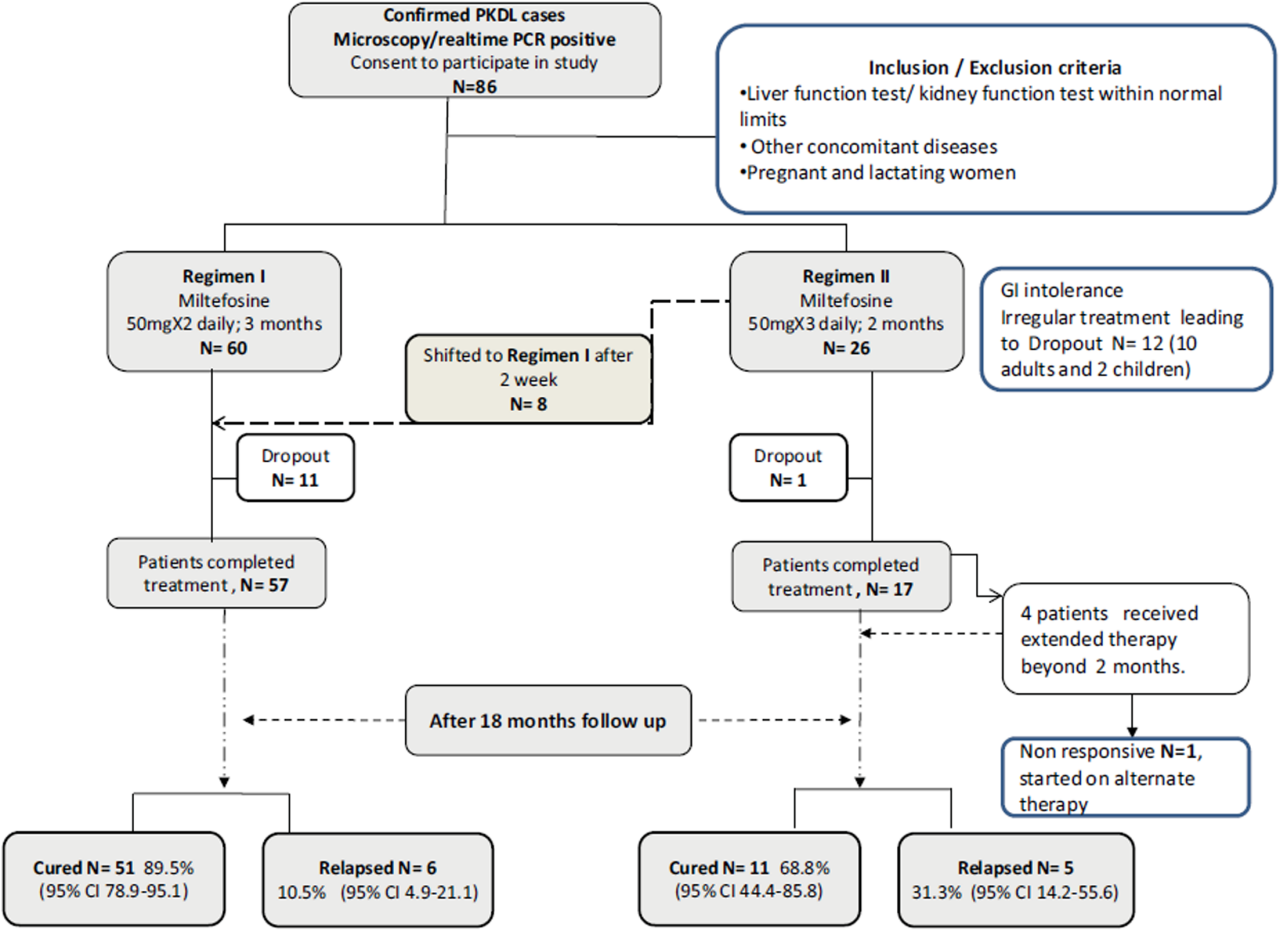
Recruitment, treatment and follow-up of PKDL patients.

Cases of PKDL (females n = 22 and males n = 64) originating from the states of Bihar, Eastern UP, Jharkhand or West Bengal that reported to Department of Dermatology, Safdarjung Hospital (SJH), New Delhi over a period of 5 years between January 2008 to December 2012 were included in this study. Baseline investigations included a complete haemogram, chest X-ray, liver and renal function tests. Patients suffering from any other infectious disease or seropositive for human immunodeficiency virus, as well as pregnant or breast feeding women were excluded from the study. PKDL diagnosis, suspected on the basis of clinical examination and positive immuno-chromatographic rK39 strip test, was confirmed by demonstration of Leishman Donovan bodies by direct microscopy of tissue/slit smear, and/or real time PCR [1,8]. Patients presented with nodular, papular or macular lesions affecting primarily the face, neck and trunk. Confirmed PKDL cases (positive for real time PCR/ microscopy) were prescribed treatment with oral MIL (Paladin Labs Inc, Montreal, Canada),50 mg twice daily for 3 months (n = 56) and 2.5mg/kg/day for 3 months in children (n = 4) referred as regimen I or 50 mg thrice daily for 2 months (n = 26) for adults referred as regimen II (Fig 1) [8]. In the beginning all the patients were advised to take regimen II of high dosage and short duration. Eight patients in regimen II who experienced frequent gastro-intestinal side-effects were switched to regimen I of lower dosage and longer duration and were considered in regimen I for analysis. Thereafter, from the year 2011, all the patients were prescribed regimen I. The patients were provided the supply of MIL for 2 weeks at a time to ensure compliance by interview before giving further supply of the drug. Liver (SGOT and SGPT) and renal function (blood urea and serum creatinine) tests were done fortnightly and found largely within the normal limits. Toxicity was graded according to the common toxicity criteria of the National Cancer Institute (http://ctep.cancer.gov/protocol/Development/electronic-applications/ctc.html as described earlier [8]. The major side effects reported were anorexia and vomiting. On the whole the symptoms were mild and the patients were able to tolerate the drug. A few patients (n = 6), however, were advised to take tablet Ondansetron (4mg) half an hour prior to taking MIL to reduce the risk of vomiting.

All the females of child bearing age were explained the consequences of becoming pregnant during the treatment and beyond. Females and their partners were repeatedly counseled to use contraceptives. Additionally, all were monitored every month for pregnancy using urine based hCG analysis. Evaluating the efficacy of treatment for predominantly macular lesions clinically is not straight forward because re-pigmentation of lesions may take place long after parasite clearance [1]. Clinical outcome was assessed monthly till the end of treatment and at 6, 12 and 18 months after treatment, using the case definition as described in Table 1. Tissue biopsy and slit aspirate samples were collected from the patients at the time of first presentation, after 1 month of treatment in clinically non-responsive cases and at one month post treatment for parasite culture and determination of parasite load as described elsewhere [9]. The patient was considered finally cured based on clinical evaluation indicated by total regression of papules/ nodules, no new lesion and near total repigmentation of maculae. In the event of reappearance of lesions, repeat histopathology, tissue biopsy culture and quantitative real time PCR were carried out to confirm relapse.

**Table 1:** Case definition for recording treatment outcome of PKDL patients.

**Early treatment outcomes**	
Initial cure	Treatment completed, clinical regression (total regression of papules/ nodules with no new lesion for polymorphic PKDL and lack of appearance of new lesions in macular PKDL), parasitological cure indicated by a negative smear and/or insignificant parasite burden by real time PCR at one month post MIL therapy
Non-responsive	Signs and symptoms of PKDL persist + a positive smear/ detectable parasite in real-time PCR at 30 days of MIL therapy.
Irregular	PKDL patients who did not take regular treatment of MIL for 90 days and/or did not present for assessment after treatment.
Extended treatment	Additional 2 to 4 weeks extended therapy in patients showing partial regression of the disease with persistence of clinical activity in the form of incompletely subsided plaques or nodules.
Alternate treatment	AmB/ Ambisome in cases of nonresponsive patients and patients with side effects requiring discontinuation of MIL
**Late treatment outcomes**	
Final cure	PKDL case with initial cure and no clinical signs (total regression of papules/ nodules, no new lesion and near total repigmentation of maculae) at 18 months after completion of therapy.
Relapse case	Cases with initial cure but with reappearance of clinical symptoms and/or signs along with smear positive for LD bodies/ parasite in real-time PCR during the 18 month follow-up.

### Parasite culture

Skin biopsy samples were collected under aseptic conditions for isolation of parasite from dermal lesions of PKDL. The epidermis was carefully dislodged and the dermal portion of the biopsy material was placed in culture medium comprising of M199 and 25mM Hepes (pH 7.4), supplemented with a mixture of vitamins and amino acids (Sigma Aldrich) and 10% heat inactivated fetal calf serum. Antibiotics including streptomycin (100 μg/ml) and penicillin (100 U/ml) were added to the medium to prevent bacterial contamination, and the samples were incubated at 26°C in a BOD incubator [6].

### Miltefosine susceptibility assay

Drug susceptibility of the parasites towards MIL was estimated at promastigote as well as amastigote stages as described previously [10, 11]. The results were expressed as the percentage reduction in the parasite viability compared to that in untreated control wells, and the 50% inhibitory concentration (IC_50_) was calculated using sigmoidal regression analysis. All experiments were performed at least thrice in quadruplicates at promastigote stage and in duplicates at amastigote stage.

### DNA isolation and parasite load determination

DNA was isolated from slit aspirate sample using QIAamp DNA isolation kit according to the manufacturer’s instructions. *Leishmania* genus-specific quantitative real-time PCR (Q- PCR), based on SYBR Green I method, was used for determination of parasite load, with. cycling parameters and primers as described previously [9]. A standard curve was constructed by 10-fold serial dilution of *L*. *donovani* DNA corresponding to 10^4^ to 0.1 parasite per reaction, plotting the Ct values against the concentration of parasite DNA and used for determination of parasite load. Each real time PCR reaction was carried out in triplicate. Results were expressed as the number of *Leishmania* parasites present in 1 μl slit aspirate.

### Statistical analysis

Statistical analysis of data was performed using Graph Pad Prism 5 software (GraphPad Software, Inc., San Diego, CA, USA). Statistical significance was determined by Mann-Whitney test and t test. P values ≤ 0.05 were considered significant.

## Results

### Clinical results

From January 2008 to Dec 2012, 86 confirmed cases of PKDL were enrolled in this study, including 82 adults and 4 children (8–12 years). Histopathology showed that the dermal infiltrate was arranged in distinct patterns depending on the type of lesion. The maculae showed mild to moderate superficial perivascular inflammation composed of lymphocytes, histiocytes and plasma cells, while the infiltrate was dense occupying the superficial and deep dermis in biopsies from papules and nodules. Less commonly, foamy change in the histiocytes and granulomas composed of epithelioid cells were seen mimicking those seen across the leprosy spectrum. Out of 86 patients who were recruited for MIL treatment, 73 (57 in Regimen I and 16 in Regimen II) completed the treatment and achieved cure. Twelve patients failed to report regularly and 1 patient was unresponsive to MIL; these 13 patients were not included for further analysis (Fig 1).

PKDL patients generally showed normal liver and renal functions during therapy, unlike VL patients [12]. Towards the end of therapy, only 3 of 73 patients in this report showed elevated SGOT (AST) and SGPT (ALT) up to 55 units/ml in comparison with the normal value of 40 units/ml. The values pertaining to kidney function tests showed no abnormality.

At the end of the treatment, a marked improvement was noticed in all the 73 patients based on the criteria defined in Table 1. Mucosal lesions were the first to regress, followed by nodules while macular lesions took the longest time to show improvement. Initial cure was achieved in seventy patients (57 from regimen I and 13 from regimen II). 56 of 59 polymorphic PKDL cases achieved initial cure with total regression of lesions at the end of the treatment. The remaining 3 patients with extensive lesions were cured only after extended treatment beyond 2 months at 150mg/day, 2 of these were treated for additional 15 days while 1 for 30 days. Likewise, all 14 macular cases were considered cured as they showed no new lesions, the only clinically appreciable sign to assess the effect of therapy. The cure rate for regimen I was 89.5% (95%C I = 78.9–95.1) and for regimen II was 68.8% (95%CI = 44.4–85.8%). In the 12 month follow up period, 3 of the 73 patients showed signs of recurrence of the disease, one at 5 months and another two at 8 months, while the remaining 70 patients completed 12 months follow up without signs of relapse. By the end of 18 months follow-up, yet another 8 patients showed signs of relapse. The profile of patients who were enrolled, completed the treatment and relapsed in this study, is described in Table 2.

**Table 2:** Profile of patients treated with miltefosine and relapsed cases.*

Variable	Total Number	Cured	Relapse (% of total treated)
Cases assigned	86		-
Cases treated	73	62 (85)	11 (15)
Male	54	46 (85)	8 (15)
Female	19	16(84)	3 (16)
Polymorphic lesions	59	51(86)	8 (14)
Macular lesions	14	11(79)	3 (21)
Cases with History of VL	54	46(85)	8 (15)
Cases treated with different drugs during VL	54	SAG- 42 (78)	SAG- 6 (11)
		AmB- 4(7)	AmB-1(2)
		MIL- 0	MIL-1(2)
Cases in HR region	51	43(84)	8 (16)
Cases in MR/LR region	19/3	16/3(84/100)	3/0 (16/0)

* Total cases recruited were 86 of which 73 completed treatment since 12 dropped out and 1 was unresponsive to MIL therapy.

Abbreviations: HR, hyper-endemic areas; MR, meso-endemic areas; LR, low-endemic areas

Eight of the 11 cases that relapsed reported a previous history of VL for which they were treated with SAG (n = 6), AmB (n = 1) or MIL (n = 1), while the patients that remained cured were treated with SAG(n = 42) or AmB(n = 4) [Table 2]. The relapses were confirmed through slit skin smear examination by microscopy and Q- PCR. Out of the 11 cases that relapsed by 18 months follow-up, 8 (73%) originated from hyperendemic region and 3 (27%) from mesoendemic region of Bihar, figures comparable to the areas of origin of the cases that did not relapse [hyperendemic = 51/73 (70%), mesoendemic region = 19/73 (26%)][7, 13]. The origin of PKDL patients from different states and endemicity zones in Bihar, UP, West Bengal and Jharkhand is shown in S1 Fig.

**Fig 2:**
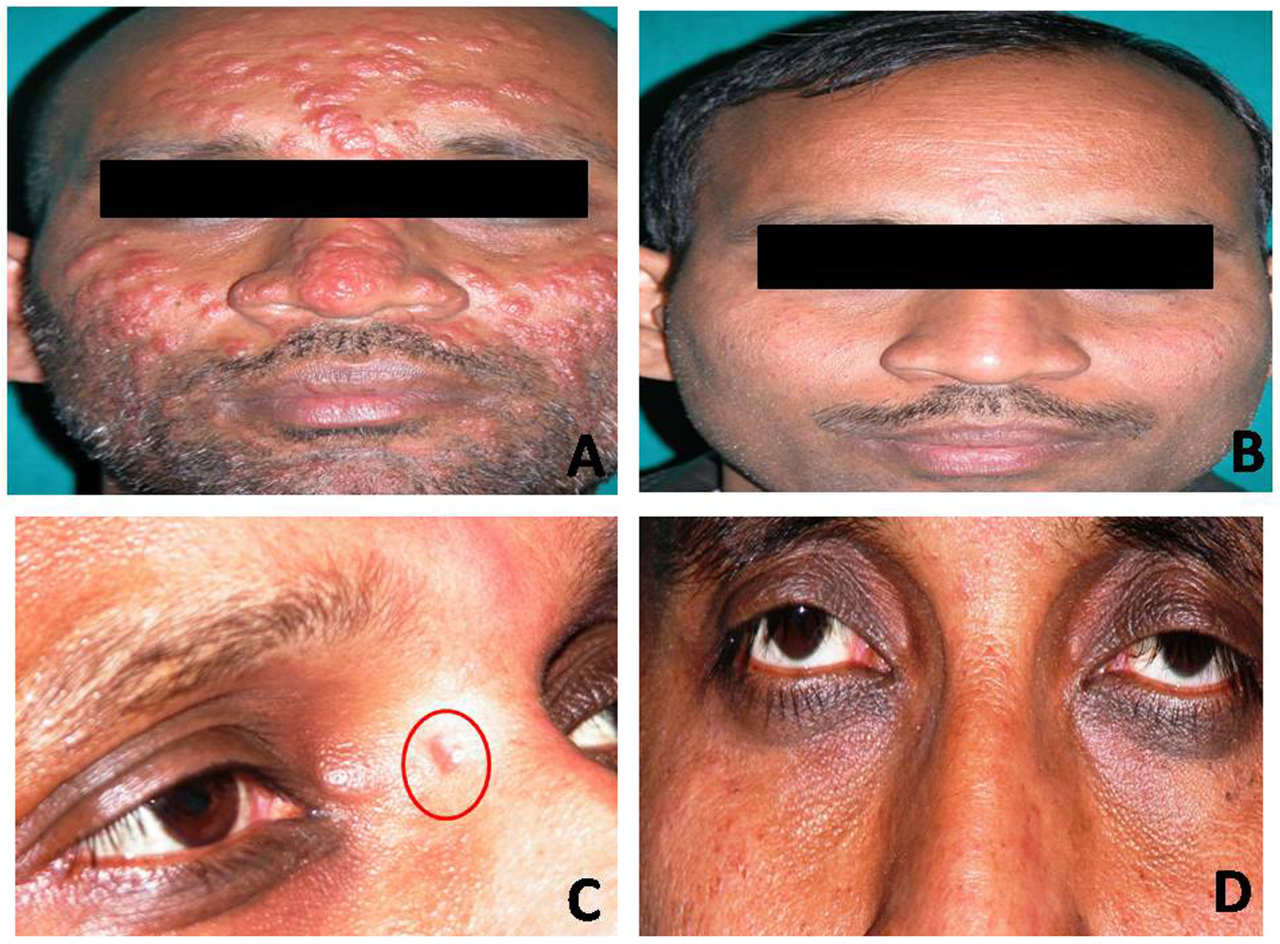
A PKDL patient (Case 11, table no. 3) that relapsed after MIL treatment and was subsequently cured with AmB. Papulo-nodules present on face before treatment (A), and complete regression after 3 months of MIL treatment (B), relapse at 13 months after completion of MIL treatment with papules on the root of nose (C), and cured after treatment with AmB (D).

The profile and presentation of cases that relapsed, along with details of treatment rendered and the parasite load, are given in S1 Table. The relapse rate at 12 month follow up was 4.1% (3/11), while it increased to 15% (8/11) at 18 months follow up. A total of 5/16 (31.3%; *95% CI 14*.*2–55*.*6*) patients relapsed after treatment in regimen II compared to 6/57 (10.5%; *95% CI 4*.*9–21*.*1*) patients in regimen I. The relapse rates in the two regimens were significantly different (p = 0.0406), indicating better efficacy of regimen I with cure rate of 89.5% (*95% CI 78*.*9–95*.*1*). The cases that relapsed were eventually treated with AmB (1500mg) or Ambisome(30mg/kg body wt). Two cases (Case 1 and Case 2, S1 Table), who did not agree to take intravenous injections, were started on a second course of MIL, however, they came back again with relapse (referred to as double relapse) and were eventually treated with AmB. A representative case of PKDL (Case 11, S1 Table) that relapsed after MIL treatment and was subsequently cured with AmB is shown in Fig 2. Importantly, there were no further relapses at 24 months of follow-up.

### Parasite load in final cured and relapse cases

**Fig 3:**
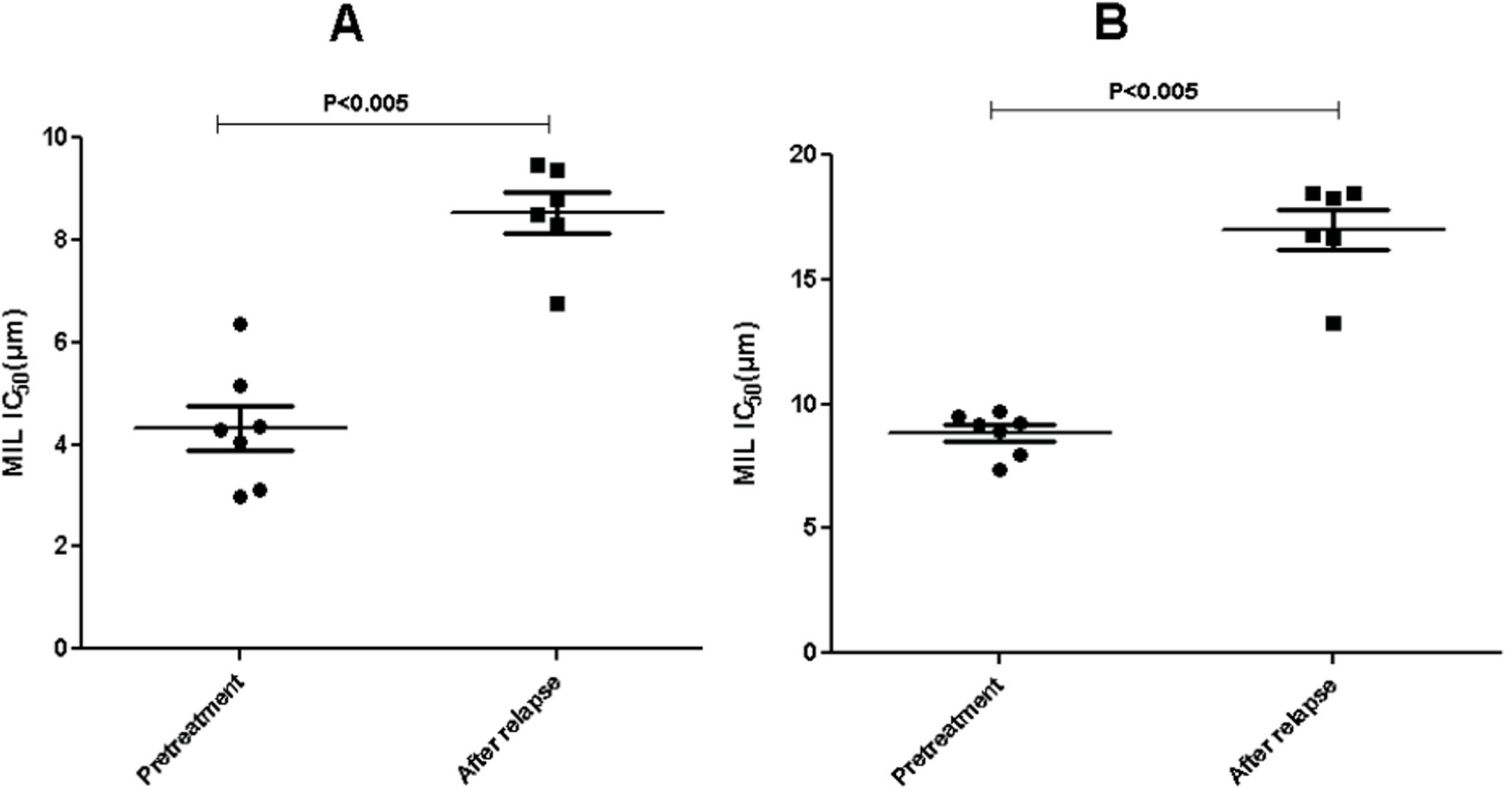
Scatter plot showing parasite load at the pre-treatment stage in the cases that eventually relapsed vs those that remained cured. Parasite load was determined by Q-PCR in slit aspirate sample at the time of diagnosis of PKDL and expressed as the number of *Leishmania* parasite/μl slit aspirate. P value was calculated using Mann-Whitney test. Horizontal bars indicate mean± SEM.

Parasite load at the pre- and post- treatment stages and at the time of relapse was determined by Q-PCR. At the pre-treatment stage, parasite load was found to be significantly higher (P<0.005) in the cases that eventually relapsed (n = 11, mean = 11,842 parasite load/ μl slit aspirate) compared to those that attained final cure (n = 62, mean = 2,302 parasite load/μl slit aspirate) (Fig 3). For the assessment of cure, parasite load was determined in a few samples (n = 30) at one month post treatment. No detectable parasites were found in slit aspirate in majority of the cases (26/30); residual parasite (< 10 parasite load/μl slit aspirate) was seen in 2 cases which relapsed and in another 2 cases which did not relapse during 18 months follow up, suggesting that the presence of residual parasite at one month post treatment may not be predictive of relapse.

### In vitro miltefosine susceptibility assay

**Fig 4:**
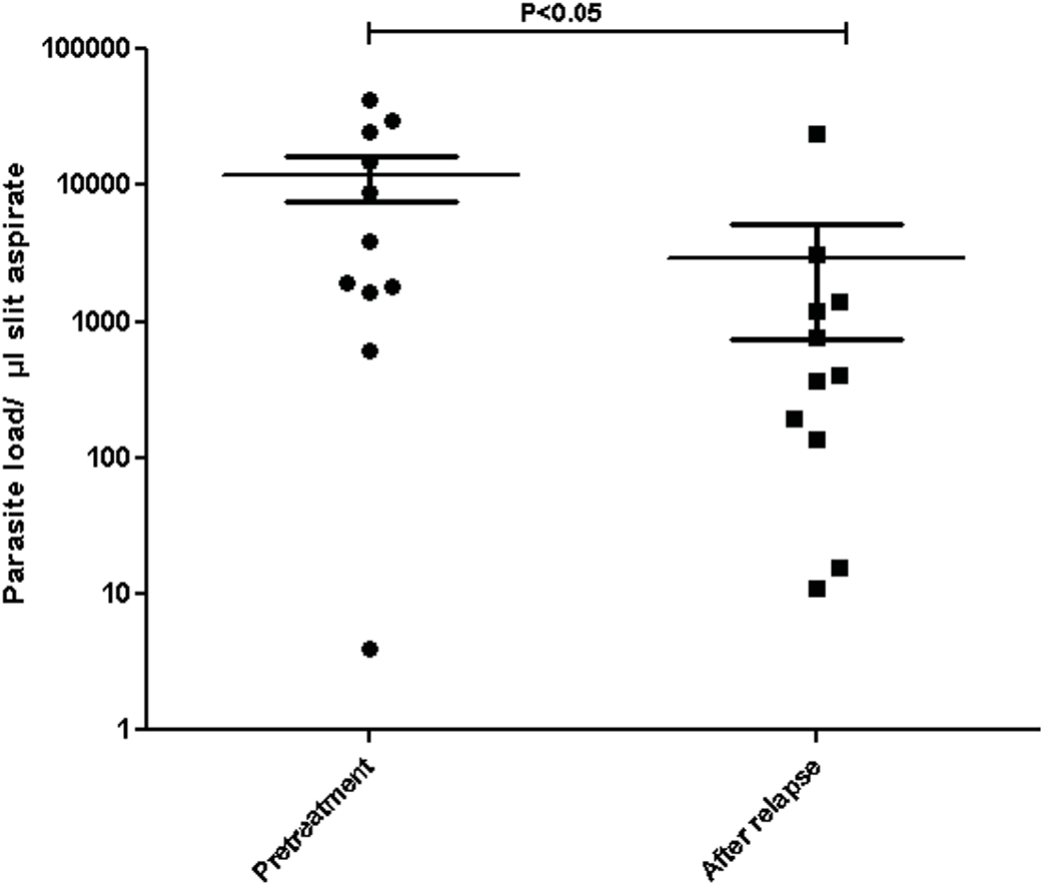
*In vitro* MIL susceptibility of parasite isolates from cured (n = 7) and relapsed (n = 6) PKDL patients. MIL susceptibility at (A) promastigote stage (B) amastigote stage. Each individual value represents mean IC_50_± SD of the results from two separate assays. P value was calculated using Mann-Whitney test. Horizontal bars indicate mean ±SEM.

MIL susceptibility was determined both at promastigote and intracellular amastigote stage for six cases that relapsed, including two double relapses, and compared with that of the pre-treatment isolates. Pre-treatment isolates showed a mean IC_50_ = 4.07±1.36 μM (range 2.27 ± 0.01 μM to 6.37 ± 1.44 μM) whereas isolates from relapse cases showed significantly (P<0.005) reduced sensitivity (mean IC_50_ = 8.53±0.98, range 6.76 ± 1.77 μM to 9.47±1.13 μM) (Fig 4A). Similarly at intracellular amastigote stage, the mean IC_50_ of pre-treatment isolates was 7.85±1.66μM (range 5.65 ± 0.7 μM to 9.6 ± 0.89 μM) which was significantly lower (P<0.005) than that of isolates from cases that relapsed (mean IC_50_ = 16.99±2.0, range 13.26±0.89 μM to 18.5±1.1 μM) (Fig 4B). We observed increasing tendency in IC_50_ of isolates from double relapse cases (18.35 ± 0.13μM) as compared to the isolates after single relapse cases (14.98 ± 2.43μM) although this difference was not significant (P = 0.18).

## Discussion

Oral MIL, introduced in India in 2002, provided a breakthrough in treating VL and was subsequently recommended for PKDL at the dosage of 50 mg twice daily for 12 weeks in adults and according to body weight in children [2, 8]. The present study was conducted to evaluate the efficacy of MIL for treatment of PKDL in 86 patients recruited over a period of five years from 2008–2012, with follow up of 18 months. Twenty-six of the 86 patients treated in the present study have been reported in our earlier study [8] which showed that, owing to side-effects such as vomiting, the duration of therapy in adults could not be reduced below 2 months at a dose of 50 mg thrice daily. All the 73 patients who completed treatment responded well and showed initial cure; however, 4% (3/73) of the patients relapsed during 12 months follow up period and the number increased to 15% (11/73) with 18 months of follow up period. The final cure rate of PKDL was 85%, which is similar to the trends observed for MIL therapy in VL in the Indian subcontinent including India, Nepal and Bangladesh [4, 5, 14]. These findings are of importance as in a previous study of MIL treatment in PKDL cases from 2008–10, no relapse was observed in a 12 months follow up period [8]. Understandably, relapse has occurred in HIV co-infected individuals [15] and incomplete cure has been seen with insufficient duration of therapy (50mg twice for 8 weeks) [16]. On the other hand a few patients may need extended treatment for cure while an occasional patient may be unresponsive [8].

Being a tertiary hospital, the cases that were referred or came to us had more prominent or extensive lesions unlike the macular types. In countries like Bangladesh within the Indian subcontinent, the macular form has been found to be common in field surveys of endemic districts as compared to hospital based referrals. In a recent study from endemic area in Bihar, it was suggested that VL patients treated with SAG developed nodular or polymorphic lesions compared to VL patients treated with AmB who developed macular lesions [17]. In our cohort with history of VL, 48 patients were treated with SAG while 5 were treated with Am B and one was treated with Mil during VL. However, the number of AmB treated cases in this cohort is too small to draw any conclusions.

We found a noticeable association between relapse rate and the different dosage regimens using MIL therapy. Regimen II (50 mg 3x daily for 60 days) resulted in significantly high relapse rate of 31% compare to 10.5% relapse rate with regimen I of longer duration. However, we did not observe any association between types of lesions and other factors like gender, age and previous VL history with relapse. In contrast to the situation with SAG treatment, the high treatment failure rate observed with MIL treatment in the present study is due to high relapse rate and not due to unresponsiveness. High relapse rate may be due to host factors or parasitic factors. With respect to parasitic factors in this study we compared (1) the MIL susceptibility profile of PKDL isolates obtained from patients at the pre-treatment, relapsed and the second relapsed stages and (2) the parasite load in the non-relapse / relapse group before the start of the treatment as well as after relapse.

Parasites isolated from patients after relapse displayed > 2 fold higher tolerance to MIL compared to isolates from pre-treatment stage, both at promastigote as well as amastigote stages. However the IC_50_ values of parasite isolates from relapsed cases were much lower than those shown for *in vitro* resistant *L donovani* parasite or the *L infantum* isolates originated from HIV-VL co-infected patients [18–20]. The MIL tolerant parasites observed here in relapse cases are a cause of concern as they may serve as a reservoir for circulation of drug tolerant parasites which may eventually give rise to drug resistant strains in VL.

We found high parasite load at the pre-treatment stage in the cases that eventually relapsed as compared to the cases that did not, indicating that the patients with higher initial parasitic burden are at a higher risk of relapse. Demonstration of parasite in PKDL tissue lesions by microscopy remains a challenge as observed by us and others [21,22]. In the present study, LD bodies could be visualized in about 30% of the patients in histopathology as compared to 50% in slit-skin smear. Histopathologically they were better seen in biopsies from mucosal sites like the tongue and buccal mucosa. Even from papulonodular lesions, LD bodies were seen in a few instances. Molecular detection methods like real time PCR used in this study provide definite diagnosis and quantitative assessment of parasite load that can indicate the severity of the disease as patients with higher parasite load were found akin to relapse. Evidence based assessment of cure in PKDL is not well established, and classical methods used for grading of severity of disease as well as regression of lesions in the diseases prognosis after treatment in cutaneous diseases like leprosy are not of much use in PKDL. The findings from this study suggest that highly sensitive and specific quantitative methods like real time PCR should be used, whenever possible, as a tool for monitoring of parasite load along with patterns of histopathological changes in the tissue for diagnosis as well as to establish relapse.

It has been shown previously that anthroponotic transmission of VL via PKDL patients may be responsible for spreading *L*. *donovani* strains that are refractory to treatment with SAG [7]. In the case of MIL the development of resistance had remained a bugbear from its inception because of its long half-life of 7 days [23]. Our findings indicate that even after effective cure of PKDL with MIL, there is a possibility of persistence of parasites in the skin. Such residual parasites which show higher tolerance towards MIL may lead to relapse of the disease and serve as a reservoir of MIL tolerant parasites.

Prolonged treatment, parenteral administration and growing resistance to antimonials had paved the way for the oral drug MIL in PKDL. The present study suggests the requirement of a close surveillance of MIL susceptibility in both VL and PKDL patients in the field. There is a need to explore molecular markers to identify drug resistance at an early stage of infection in order to help designing treatment strategies to eliminate this infectious disease. This report demonstrates that the efficacy of monotherapy with MIL for PKDL has reduced to 85%, indicating the need for other drugs/ combination therapy with MIL.

## Supporting Information

S1 Fig Map of Bihar and the adjoining states showing the origin of PKDL patients from regions of different endemicity. Patients hailed from the states of Bihar (N = 80), Uttar Pradesh (N = 4), West Bengal (N = 1) and Jharkhand (N = 1).
(TIF)

S1 Table Profile, presentation, parasite load and treatment details in PKDL patients that relapsed. (DOCX)

## References

[1] World Health Organization (WHO). Post kala-azar dermal leishmaniasis (PKDL): A manual for case management and control. Report of a WHO consultative meeting. Kolkata, India 2012; 6–13.

[2] Sundar S, Jha TK, Thakur CP, Engel J, Sindermann H, Fischer C, et al Oral miltefosine for Indian visceral leishmaniasis. N Engl J Med 2002; 347:1739–1746. 12456849 10.1056/NEJMoa021556

[3] Sundar S, Sinha P, Jha TK, Chakravarty J, Rai M, Kumar N, et al Oral miltefosine for Indian post kala-azar dermal leishmaniasis: a randomised trial. Trop Med Int Health 2013; 18:96–100. 10.1111/tmi.12015 23136856

[4] Sundar S, Singh A, Rai M, Prajapati VK, Singh AK, Ostyn B, et al Efficacy of miltefosine in the treatment of visceral leishmaniasis in India after a decade of use. Clin Infect Dis 2012; 55:543–550. 10.1093/cid/cis474 22573856

[5] Rijal S, Ostyn B, Uranw S, Rai K, Bhattarai NR, Dorlo TP, et al Increasing failure of miltefosine in the treatment of Kala-azar in Nepal and the potential role of parasite drug resistance, reinfection, or noncompliance. Clin Infect Dis 2013; 56: 1530–1538. 10.1093/cid/cit102 23425958

[6] Bhandari V, Kulshrestha A, Deep DK, Stark O, Prajapati VK, Ramesh V, et al Drug susceptibility in *Leishmania* isolates following miltefosine treatment in cases of visceral leishmaniasis and post kala-azar dermal leishmaniasis. PLoS Negl Trop Dis 2012;6:e1657 10.1371/journal.pntd.0001657 22629478 PMC3358331

[7] Singh R, Kumar D, Ramesh V, Negi NS, Singh S, Salotra P. Visceral leishmaniasis, or kala azar (KA): high incidence of refractoriness to antimony is contributed by anthroponotic transmission via post-KA dermal leishmaniasis. J Infect Dis 2006; 194:302–306. 16826477 10.1086/505079

[8] Ramesh V, Katara GK, Verma S, Salotra P. Miltefosine as an effective choice in the treatment of post kala-azar dermal leishmaniasis. Br J Dermatol 2011; 165:411–414. 10.1111/j.1365-2133.2011.10402.x 21561437

[9] Verma S, Bhandari V, Avishek K, Ramesh V, Salotra P. Reliable diagnosis of post kala-azar dermal leishmaniasis (PKDL) using slit aspirate specimen to avoid invasive sampling procedures. Trop Med Int Health 2013; 18:268–275. 10.1111/tmi.12047 23279800

[10] Kulshrestha A, Bhandari V, Mukhopadhyay R, Ramesh V, Sundar S, Maes L, et al Validation of a simple resazurin-based promastigote assay for the routine monitoring of miltefosine susceptibility in clinical isolates of *Leishmania donovani* . Parasitol Res 2013; 112:825–828. 10.1007/s00436-012-3212-3 23239091

[11] Bhandari V, Kumar D, Verma S, Srividya G, Negi NS, Singh R, et al Increased parasite surface antigen-2 expression in clinical isolates of *Leishmania donovani* augments antimony resistance. Biochem Biophys Res Commun 2013; 440:646–651. 10.1016/j.bbrc.2013.09.113 24103752

[12] Bhattacharya SK, Sinha PK, Sundar S, Thakur CP, Jha TK, Pandey K et al Phase 4 trial of miltefosine for the treatment of Indian visceral leishmaniasis. J Infect Dis 2007; 196:591–598. 17624846 10.1086/519690

[13] Croft SL, Sundar S, Fairlamb AH. Drug resistance in leishmaniasis. Clin Microbiol Rev. 2006;19:111–126. 16418526 10.1128/CMR.19.1.111-126.2006 PMC1360270

[14] Rahman M, Ahmed BN, Faiz MA, Chowdhury MZ, Islam QT, Sayeedur R, et al Phase IV trial of miltefosine in adults and children for treatment of visceral leishmaniasis (kala-azar) in Bangladesh. Am J Trop Med Hyg 2011; 85:66–69. 10.4269/ajtmh.2011.10-0661 21734127 PMC3122346

[15] Ramesh V, Avishek K, Salotra P. Postkala-azar dermal leishmaniasis in HIV-coinfected individuals: problems in diagnosis and treatment. Int J Dermatol. 2015; 54:116–120. 10.1111/ijd.12665 25209701

[16] Khandpur S, Chaturvedi P, Kumar U, Khaitan BK, Samantaray JC, Sharma VK.Oral miltefosine in post kala-azar dermal leishmaniasis. Int J Dermatol 2010; 49:565–569.17. 10.1111/j.1365-4632.2010.04326.x 20534094

[17] Burza S, Sinha PK, Mahajan R, Sanz MG, Lima MA, Mitra G, et al Post kala-azar dermal leishmaniasis following treatment with 20 mg/kg liposomal amphotericin B (Ambisome) for primary visceral leishmaniasis in Bihar, India. PLoS Negl Trop Dis 2014: 8: e2611 10.1371/journal.pntd.0002611 24392171 PMC3879248

[18] Cojean S, Houzé S, Haouchine D, Huteau F, Lariven S, Hubert V, et al *Leishmania* resistance to miltefosine associated with genetic marker. Emerg Infect Dis 2012; 18:704–706. 10.3201/eid1804.110841 22469394 PMC3309694

[19] Cojean S, Houzé S, Haouchine D, Huteau F, Lariven S, Hubert V, et al Inactivation of the miltefosine transporter, LdMT, causes miltefosine resistance that is conferred to the amastigote stage of *Leishmania donovani* and persists *in vivo* . Int J Antimicrob Agents 2007; 30:229–235. 17628445 10.1016/j.ijantimicag.2007.05.007

[20] Kulshrestha A, Sharma V, Singh R, Salotra P. Comparative transcript expression analysis of miltefosine-sensitive and miltefosine-resistant *Leishmania donovani* . Parasitol Res 2014; 113:1171–1184. 10.1007/s00436-014-3755-6 24449447

[21] Rathi SK, Pandhi R K, Chopra P, Khanna N. Post-kala-azar dermal leishmaniasis: A histopathological study. Indian J Dermatol Venereol Leprol 2005; 71:250–253. 16394433 10.4103/0378-6323.16616

[22] Singh N, Ramesh V, Arora VK, Bhatia A, Kubba A, Ramam M. Nodular post-kala-azar dermal leishmaniasis: a distinct histopathological entity. J Cutan Pathol 1998; 25:95–99. 9521498 10.1111/j.1600-0560.1998.tb01696.x

[23] Berman J. Miltefosine to treat leishmaniasis. Expert Opinion Pharmacother 2005; 6: 1381–1388. 10.1517/14656566.6.8.1381 16013987

