# Correction: Decline in Clinical Efficacy of Oral Miltefosine in Treatment of Post Kala-azar Dermal Leishmaniasis (PKDL) in India

**DOI:** 10.1371/journal.pntd.0004289

**Published:** 2015-12-07

**Authors:** V. Ramesh, Ruchi Singh, Kumar Avishek, Aditya Verma, Deepak Kumar Deep, Sandeep Verma, Poonam Salotra

Fig 3 and Fig 4 are incorrect. The authors have provided corrected versions here.

**Fig 3:**
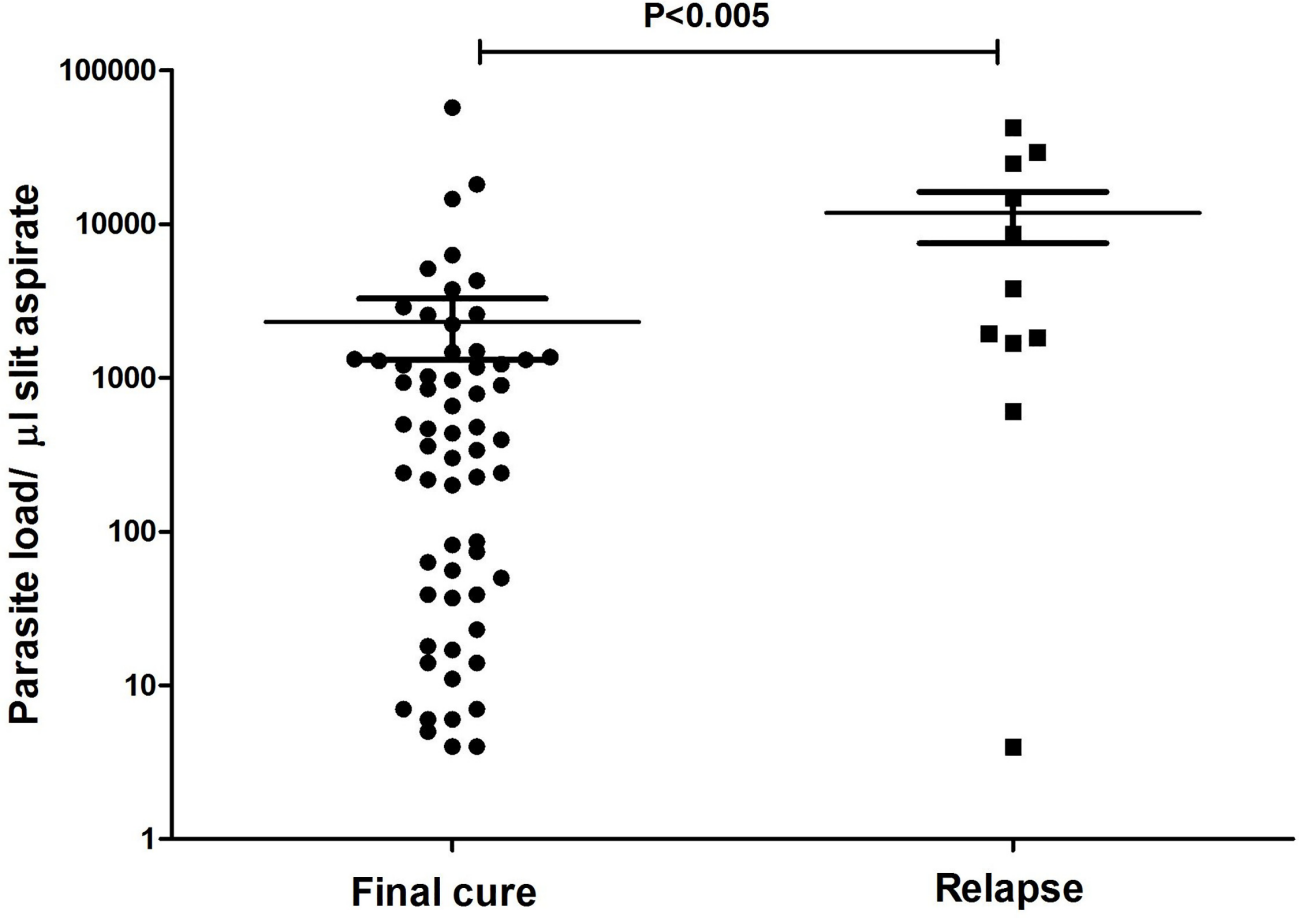
Scatter plot showing parasite load at the pre-treatment stage in the cases that eventually relapsed vs those that remained cured. Parasite load was determined by Q-PCR in slit aspirate sample at the time of diagnosis of PKDL and expressed as the number of *Leishmania* parasite/μl slit aspirate. P value was calculated using Mann-Whitney test. Horizontal bars indicate mean± SEM.

**Fig 4:**
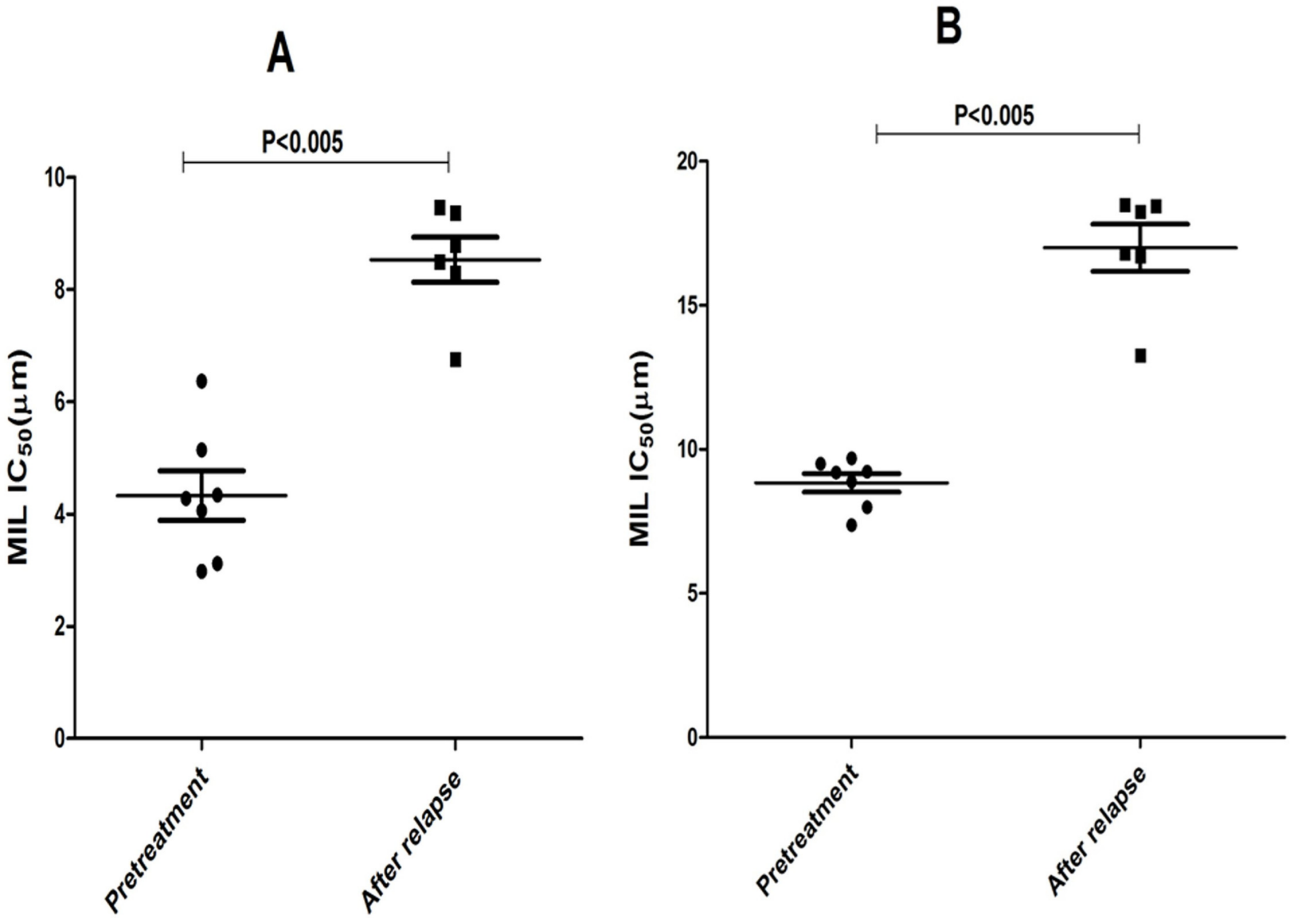
*In vitro* MIL susceptibility of parasite isolates from cured (n = 7) and relapsed (n = 6) PKDL patients. MIL susceptibility at (A) promastigote stage (B) amastigote stage. Each individual value represents mean IC_50_± SD of the results from two separate assays. P value was calculated using Mann-Whitney test. Horizontal bars indicate mean ±SEM

## References

[1] Ramesh V, Singh R, Avishek K, Verma A, Deep DK, Verma S, et al (2015) Decline in Clinical Efficacy of Oral Miltefosine in Treatment of Post Kala-azar Dermal Leishmaniasis (PKDL) in India. PLoS Negl Trop Dis 9(10): e0004093 doi:10.1371/journal.pntd.0004093 26492039 10.1371/journal.pntd.0004093 PMC4619646

