# Supplementary figures and images for: Decline in Clinical Efficacy of Oral Miltefosine in Treatment of Post Kala-azar Dermal Leishmaniasis (PKDL) in India
Source: PLoS Negl Trop Dis. 2015 Oct 22;9(10):e0004093. doi: 10.1371/journal.pntd.0004093 (PMC4619646; doi:10.1371/journal.pntd.0004093)

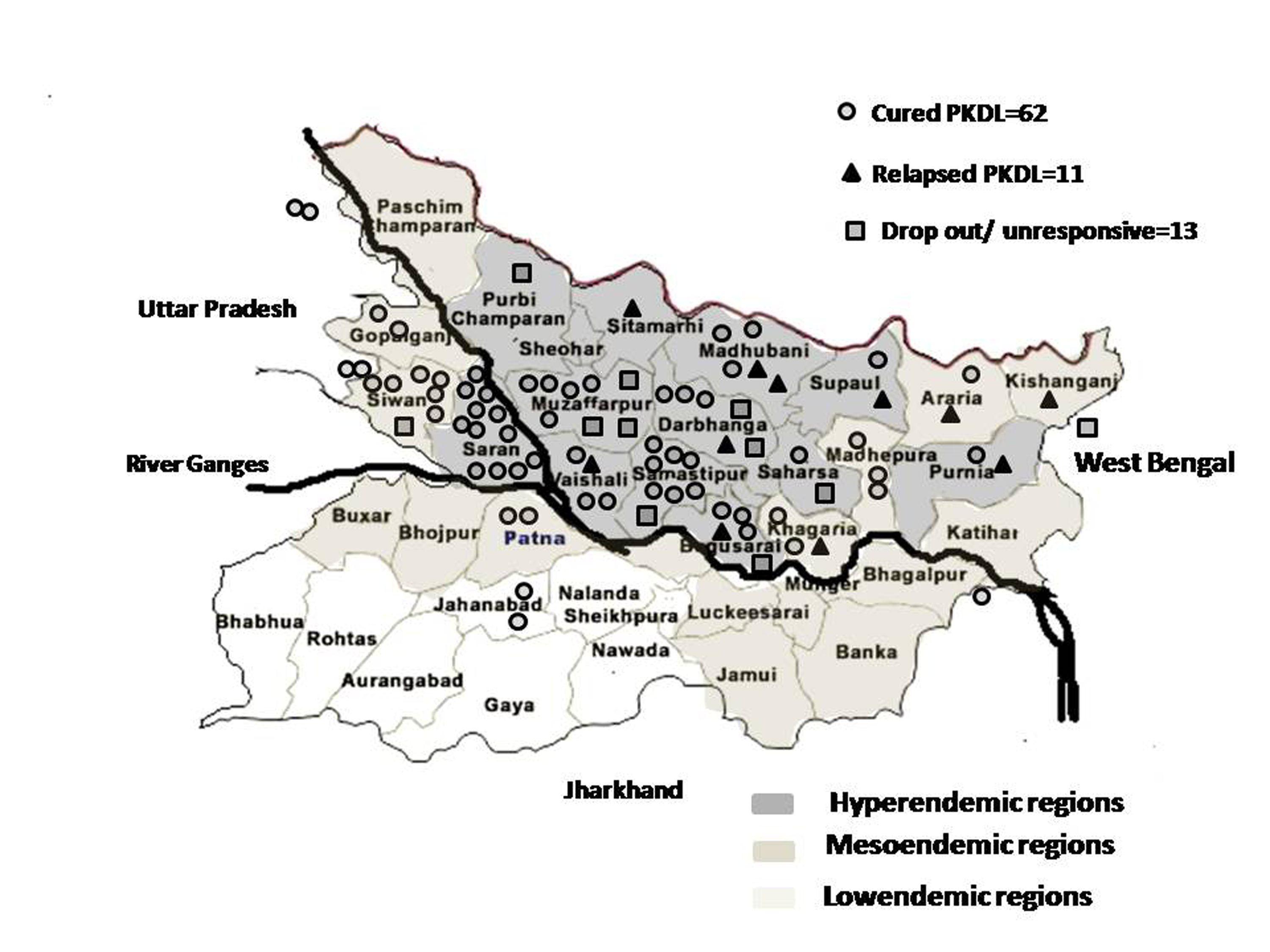

Supplement: S1 Fig — Patients hailed from the states of Bihar (N = 80), Uttar Pradesh (N = 4), West Bengal (N = 1) and Jharkhand (N = 1). (TIF) [file pntd.0004093.s001.tif]
