# Supplementary material for: Decline in Clinical Efficacy of Oral Miltefosine in Treatment of Post Kala-azar Dermal Leishmaniasis (PKDL) in India
Source: PLoS Negl Trop Dis. 2015 Oct 22;9(10):e0004093. doi: 10.1371/journal.pntd.0004093 (PMC4619646; doi:10.1371/journal.pntd.0004093)
Supplement: S1 Table — (DOCX) [file pntd.0004093.s002.docx]

**Table S**: Profile, presentation, parasite load and treatment details in PKDL patients that relapsed

| **S.No** | **Age/ Sex** | **Year of recruit-ment** | **Presentation** | **Miltefosine**  **treatment*** | **Time of relapse#**  ( Months) | **Parasite load /μl slit aspirate** | | |
| --- | --- | --- | --- | --- | --- | --- | --- | --- |
|  |  |  |  |  |  | Before treatment | Post treatment | At relapse**^$^** |
| 1 | 34/m | 2008 | P, N | Thrice daily, 75 d | 13 | 3792 | Nil | 3058 |
| 2 | 23/m | 2009 | M ,P,N | Thrice daily, 75 d | 15 | 42460 | Nil | 1400 |
| 3 | 19/m | 2011 | Eryth M | Thrice daily, 60 d | 12 | 1680 | NA | 407 |
| 4 | 20/f | 2011 | Eryth M | Twice daily, 90d | 12 | 24805 | NA | 365 |
| 5 | 19/m | 2011 | P,N | Thrice daily, 60 d | 8 | 14870 | 5 | 1217 |
| 6 | 23/m | 2011 | M,P,N | Thrice daily, 60 d | 14 | 1938 | Nil | 136 |
| 7 | 28/f | 2011 | Eryth M | Twice daily, 90 d | 15 | 18 | NA | 11 |
| 8 | 35/m | 2012 | Eryth M, N | Twice daily, 90 d | 8 | 29599 | NA | 24107 |
| 9 | 47/m | 2012 | M,P,N | Twice daily, 90 d | 5 | 8679 | NA | 191 |
| 10 | 27/m | 2012 | M,P | Twice daily, 90 d | 15 | 1824 | 7 | 16 |
| 11 | 38/m | 2012 | P,N | Twice daily, 90 d | 13 | 604 | NA | 777 |

* 50 mg of MIL per dose.

**#** Months post treatment

$ All skin slits were LD negative by microscopy at relapse.

**Abbreviations:** m, male; f, female**;** M, Macules; P, Papules; N, Nodules; Eryth M, Erythematous macules; d, days; NA, not available.
